# Schizophrenia and Multiple Sclerosis: Common pathways, common risk-factors

**DOI:** 10.1192/j.eurpsy.2022.2036

**Published:** 2022-09-01

**Authors:** H. Jemli, U. Ouali, S. Madouri, A. Aissa, R. Jomli

**Affiliations:** 1 university of tunis elmanar, Faculty Of Medicine Of Tunis, manouba, Tunisia; 2 Razi Hospital, Psychiatry A, manouba, Tunisia

**Keywords:** inflammation, multiple sclerosis, resistance, schizophrénia

## Abstract

**Introduction:**

Schizophrenia (SCZ) is a severe mental disorder that is among the leading causes of disability worldwide. Multiple sclerosis (MS) is a chronic inflammatory neurological disease with a major impact on the quality of life of young adults. Despite the distinct nature of these two disorders, research studies have identified similarities in underlying pathological mechanisms and risk factors.

**Objectives:**

To illustrate, through a case report, the central role of inflammation in schizophrenia and its relationship with multiple sclerosis.

**Methods:**

Case Report of a 31-year-old male patient with schizophrenia who has been diagnosed with multiple sclerosis.

**Results:**

Mr M. is a 31 year old patient who was diagnosed with schizophrenia at age 17. Between the ages of 25 and 27, the patient had two severe psychotic relapses each one requiring inpatient treatment. At that time, he experienced predominantly severe positive symptoms and persistent suicidality. He was initially prescribed amisulpride up to 600mg, followed by haloperidol up to 45mg daily. Due to poor clinical response, the patient was put on clozapine 400mg/d and has been stabilized since 2017, with outpatient checkups. The patient has reported vertigo and trouble walking in August 2021. He has been referred to the Neurology Department. Clinical, biological and imaging findings were highly suggestive of Multiple sclerosis and the patient has received short courses of intravenous corticosteroids.

**Conclusions:**

This case report highlights the possible association between Multiple Sclerosis and schizophrenia. Further research is needed to clarify the role of inflammation in the central nervous system in schizophrenia and the overlap with Multiple Sclerosis.

**Disclosure:**

No significant relationships.

